# Geometric pinning and antimixing in scaffolded lipid vesicles

**DOI:** 10.1038/s41467-020-17432-w

**Published:** 2020-09-04

**Authors:** Melissa Rinaldin, Piermarco Fonda, Luca Giomi, Daniela J. Kraft

**Affiliations:** 1 Huygens-Kamerlingh Onnes Lab, Universiteit Leiden, Leiden, 2300 RA The Netherlands; 2 Instituut-Lorentz, Universiteit Leiden, Leiden, 2300 RA The Netherlands; 3 Present Address: Martin A. Fischer School of Physics, Brandeis University, Waltham, MA 02453 USA; 4 Present Address: Max Planck Institute of Colloids and Interfaces, Potsdam, 14476 Germany

**Keywords:** Colloids, Organic molecules in materials science, Biological physics

## Abstract

Previous studies on the phase behaviour of multicomponent lipid bilayers found an intricate interplay between membrane geometry and its composition, but a fundamental understanding of curvature-induced effects remains elusive. Thanks to a combination of experiments on lipid vesicles supported by colloidal scaffolds and theoretical work, we demonstrate that the local geometry and global chemical composition of the bilayer determine both the spatial arrangement and the amount of mixing of the lipids. In the mixed phase, a strong geometrical anisotropy can give rise to an *antimixed* state, where the lipids are mixed, but their relative concentration varies across the membrane. After phase separation, the bilayer organizes in multiple lipid domains, whose location is pinned in specific regions, depending on the substrate curvature and the bending rigidity of the lipid domains. Our results provide critical insights into the phase separation of cellular membranes and, more generally, two-dimensional fluids on curved substrates.

## Abstract (web summary)

Lipid bilayers feature an intricate interplay between membrane geometry and its chemical composition but lack of a model system with simultaneous control over membrane shape and composition prevented a fundamental understanding of curvature-induced effects. Here the authors demonstrate that the local substrate geometry and global chemical composition of the bilayer determine both the spatial arrangement and the sorting of lipid domains.

## Introduction

Both in eukaryotes and prokaryotes, cells are capable of remodelling their lipid membranes to create a plethora of functionalities: from cargo-trafficking vesicles to division and motility^1^. These conformational changes are achieved through different biophysical strategies, including local variations in the chemical composition of the lipids, the reorganization of the actin cortex and the expression of curvature-generating proteins. In turn, the resulting modulation of local curvature serves to recruit curvature-sensing proteins and lipids, which may further stabilize or increase local curvature differences^2–5^. Recent experiments on cells subject to controlled deformation, have confirmed the primary role of spatial curvature for inducing actin polymerization and recruiting curvature sensitive proteins^6,7^.

Inspired by these observations, the correlation between membrane geometry and chemical composition has also been extensively studied in model lipid membranes consisting of ternary mixtures of phospholipids and cholesterol. While chemically homogeneous at high temperatures, below a certain temperature they spontaneously separate into two liquid phases. These phases, known as liquid-ordered (LO) and liquid-disordered (LD), differ by lipid composition and material properties, such as bilayer thickness and bending resistance^8–10^. Specifically, the LO phase is thicker and stiffer and is energetically favoured to occupy regions of low curvature, at the expense of the thinner and softer LD phase. Experiments on giant unilamellar vesicles (GUVs)^9,11–18^ reported a twofold correlation between these lipid domains and membrane geometry: while local membrane curvature can favour lipid segregation and domain nucleation and localization, the presence of lipid domains can also drive the formation of curved regions, including buds, necks, and protrusions.

To solve this causality dilemma, several experimental setups have been proposed to control membrane shape and investigate its effect on phase separation patterns. One method developed to set membrane geometry consists of pulling highly curved tubes from GUVs. These experiments showed that, close to the demixing point, the more curved the tubes the higher the concentration of unsaturated lipids in the tubes, thereby demonstrating that curvature affects the sorting of lipids^15^. Another approach consists of coating non-flat substrates with lipid bilayers. This has been achieved by bringing GUVs in contact with solid substrates^19^ or fabricating supported lipid bilayers on topographically patterned surfaces^17,18^. In both experimental setups, the LO (LD) domains were found to occupy regions of low (high) curvature.

While these experiments have greatly contributed to our understanding of the physics and chemistry of liquid-liquid phase separation in membranes, they could not disentangle the correlation between membrane geometry, lipid composition, and resulting phase separation patterns. In nonspherical GUVs, membrane shape changes during phase separation. By contrast, in SLBs where membrane geometry can be prescribed, the correlation between lipid composition and substrate geometry is elusive, because of the presence of boundaries as well as large system size, which hinders the resolution of small differences in the concentration of domains^17,18^.

In this work, we overcome these limitations by fabricating multicomponent scaffolded lipid vesicles (SLVs), consisting of lipid bilayers supported by colloidal particles. By tuning the shape of the colloids, we can obtain different membrane shapes, similar to previous work on nanoparticles^20,21^. Importantly, unlike topographically patterned surfaces, SLVs are topologically closed: i.e. their surface is compact and has no boundary. This allows decoupling the mutual interplay between spatial curvature and organization of lipid domains. Moreover, these feature also allow us to generalize the notion of curvature-induced lipid sorting by identifying states, that we term *antimixing*, where lipids are mixed and yet organized in domains having strikingly different concentrations.

## Results

### Design of multicomponent scaffolded lipid vesicles

**Fig. 1:**
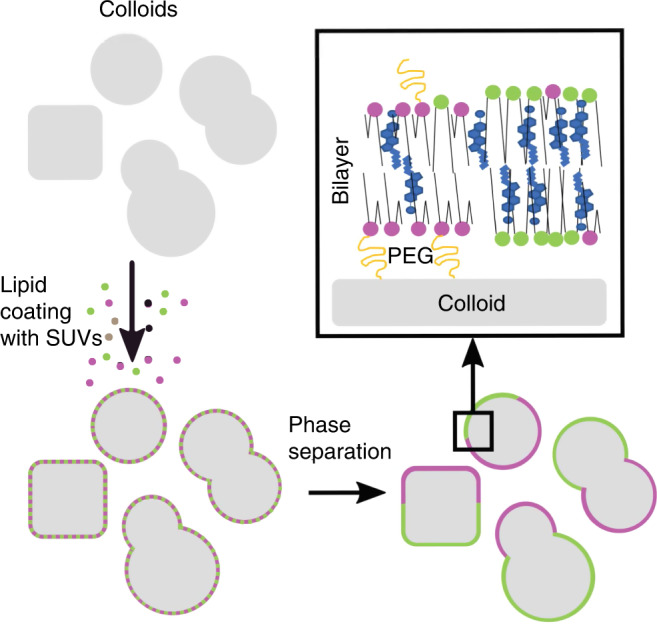
**Schematic overview of the experimental system**. Lipid coating of silica-coated particles with spherical, cubic, dumbbell, and snowman shape by small unilamellar vesicles (SUVs). After the temperature is lowered, the lipid bilayer undergoes phase separation. Liquid-disordered (LD) and liquid-ordered (LO) phases are represented in magenta and green, respectively. PEG molecules are drawn in yellow and cholesterol lipids in light blue.

**Fig. 2:**
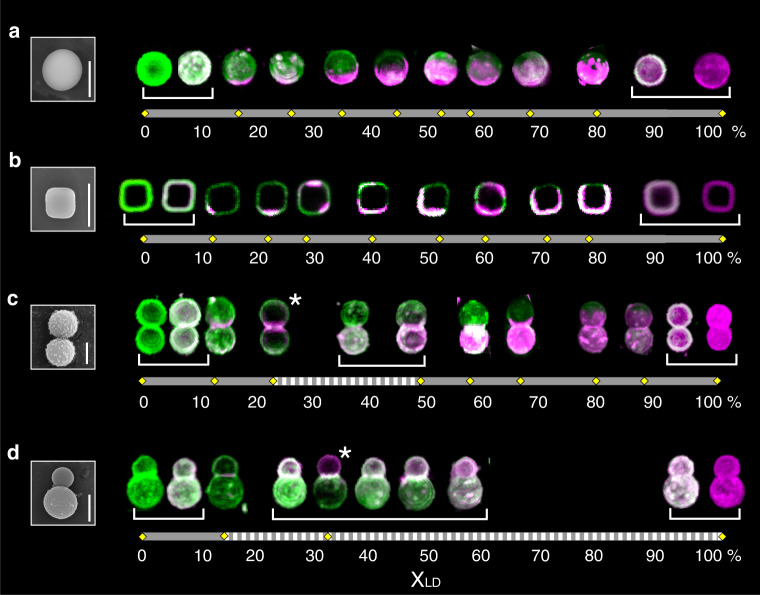
**Representative collection of segregated SLVs ordered by percentage of area occupied by the LD phase**. For each of the four different shapes considered, we order the membrane configurations by the relative surface area occupied by the POPC-rich domains. Yellow rhombuses on the horizontal bars indicate measured values of *x*_LD_. Dashed portions of the bar correspond to gaps, i.e. ranges in the area for which no configurations have been observed. Homogeneously mixed and antimixed configurations grouped with a white parenthesis correspond to the same value of *x*_LD_ and thus to a single rhombus. The left panel shows scanning electron microscopy (SEM) images of **a** spherical, **b** cubic, **c** symmetric, and **d** asymmetric dumbbell-shaped particles. The right panel shows the corresponding SLVs. For spheres and dumbbell-shaped particles, 3D reconstructions are reported. For cubic shaped particles, equatorial sections are reported. The asterisks show the two configurations that we used for Fig. 5. See Table 1 in Methods for values of percentage of area occupied by the LD phase. Scale bars are 2 μm.

We fabricate SLVs by deposition of small unilamellar vesicles (SUVs) on colloidal particles in the micrometer size range (see Fig. 1). SUVs are prepared from a ternary mixture of porcine brain sphingomyelin (BSM), 1-palmitoyl-2-oleoyl-*sn*-glycero-3-phosphocholine (POPC), and cholesterol (Chol) in a 2:1:1 mole ratio^22^ by extrusion. At this composition, free-standing lipid vesicles show phase separation, see Supplementary Fig. 5. We deposited the obtained SUVs on micron-sized particles of four shapes: spheres (Fig. 2a), cubes (Fig. 2b), symmetric (Fig. 2c), and asymmetric dumbbells (Fig. 2d), the latter are also called snowman particles in the following. See Methods and Sec. A of Supplementary Methods for brief and extensive discussion on particle syntheses, respectively. Even though only one specific molar ratio of lipids is used in the preparation of the SUVs, the single composition of any given SUV is randomly distributed around 2:1:1.

Colloidal particles attract SUVs via Van der Waals and electrostatic forces. Upon contact, SUVs burst and fuse forming a homogeneous lipid bilayer, which fully envelopes the surface^23,24^. To enhance this effect while preserving fluidity, we use colloids with a silica surface and 5% mole of 1,2-dioleoyl-*sn*-glycero-3-phosphoethanolamine-N-[methoxy(polyethylene glycol)-2000] (DOPE-PEG 2000) (top-right inset of Fig. 1), see Methods. We confirmed both the homogeneity (Supplementary Fig. 3) and the liquid nature of the membrane via fluorescence after photobleaching (FRAP) experiments (Supplementary Fig. 4 and Supplementary Methods). For more details on the lipid bilayer coating and characterization, see ref. ^24^. We stress that, upon deposition on the colloidal substrate, the lipids are well into the mixed phase as the SUVs are kept at temperatures significantly above demixing, whose critical temperature is expected to be in the range 35–45 °C^22^. This precaution ensures homogeneity in the composition of the lipid bilayers and guarantees that phase separation occurs only on the colloidal substrate, once the lipids have formed a closed bilayer.

After lipid deposition, we lower the temperature to induce in-plane lipid segregation and image the membranes using confocal microscopy. We identify the LO (BSM-rich) and LD (POPC-rich) phases through fluorescent labelling with N-[11-(dipyrrometheneboron difluoride)undecanoyl]-D-*erythro*-sphingosylphosphorylcholine (C11 TopFluor SM) and 1,2-dioleoyl-sn-glycero-3-phosphoethanolamine-N-lissamine rhodamine B sulfonyl 18:1 (Liss Rhod PE)^25^ shown, respectively, in green and magenta in Fig. 2. We emphasize that all membrane components are present in both phases, albeit at different concentrations^25–29^. We avoid changes in the chemical composition due to oxidation by preparing the SUVs via extrusion rather than sonication and keeping the sample in the dark to prevent bleaching. This fabrication process yields multicomponent lipid vesicles whose shape is scaffolded by the surface of the supporting anisotropic colloidal particles.

### Experimental results

Our multicomponent SLVs enable us to study lipid phase separation of closed surfaces with prescribed geometry. While the global mole ratio of the three lipids of the SUVs is fixed at 2:1:1 of BSM:POPC:Chol, our setup leads to the deposition of a random amount of lipids of each kind on any given SLV. This allows us to sample multiple membrane compositions in a single experiment.

We assess whether a given SLV is phase-separated or mixed by measuring the intensity of the two dyes at different locations on the surface. If the intensity signals from both dyes normalized with the maximum intensities overlap completely, the SLV is in the mixed state, otherwise it is phase-separated. For phase-separated SLVs, intensity profiles were acquired for 3D stacks of spherical- and dumbbell-shaped SLVs using confocal microscopy. Due to the small size of the cubic particles (corner-to-corner distance 1.8 ± 0.1 μm), however, the acquisition of 3D stacks was challenging and we restricted the measurement to the equatorial plane. Finally, by assigning a binary value to each pixel within an image, we measured the percentage of area occupied by each phase, which we, respectively, denote as *x*_LD_ and *x*_LO_.

We found that the likelihood of configurations, which present an interface is affected by the geometry of the substrate. By collecting data for 200 SLVs of each shape, we observed that 22% of the spheres exhibited segregated lipid domains, a value rising to 68% and 91% for symmetric and asymmetric dumbbells, respectively. These significantly different values do not stem from any differences in the type of silica of the colloid surface, see Supplementary Methods. Since all external conditions were kept identical, including the SUV composition, we conclude that geometry must promote the segregation of lipids and effectively widen the phase segregation region in the ternary phase diagram.

A simple way to organize our data for segregated SLVs is to order them by their value of *x*_LD_, i.e. by the percentage of their surface area occupied by the POPC-rich domains. This classification is inherently one-dimensional and is not directly linked to the relative amount of the three lipids on a single SLV, which would correspond to a point within the Gibbs phase triangle and to which we have no experimental access. The result of this procedure for the different geometries we have considered is shown in Fig. 2 (see also Table 1).

We observed a correlation between the underlying scaffold curvature and the size, structure, and location of phase domains. Since spheres are uniformly curved, domain equilibration dynamics is driven by interface length minimization, which systematically relaxes towards the formation of two domains bounded by a single interface (see Fig. 2a). Unlike in previous experiments on lipid bilayers on spherical colloidal particles, in which the membrane was showing multiple coexisting domains (see e.g. ref. ^30^), this observation suggests that our system has likely reached equilibrium already a few minutes after cooling. This fast equilibration has the further benefit of reducing the effect of substrate-induced drag, which, as shown in ref. ^31^, could significantly affect the coarsening dynamics of lipid domains. We emphasize that keeping the SUVs above the transition temperature during formation and coating is instrumental to these results. In control experiments with SLVs prepared from SUVs that were allowed to cool to room temperature before coating, we did not find two or three domains only, but multiple, randomly localized domains similar to results reported in ref. ^30^ (Supplementary Fig. 11). Moreover, the supported membrane appeared to have varying thickness and FRAP experiments only showed partial recovery. These observations point to incomplete fusion of SUVs on the surface, which prohibits attainment of an equilibrium state.

**Table 1:** LD area fractions of the SLVs shown in Fig. 2.

Sphere	Cube	Dumbbell	Snowman
18 ± 3	11 ± 1	13 ± 1	13 ± 2
26 ± 2	22 ± 2	23 ± 5	32 ± 2
35 ± 3	28 ± 3	50 ± 2	
45 ± 3	41 ± 2	57 ± 4	
52 ± 5	52 ± 2	67 ± 2	
58 ± 2	60 ± 2	80 ± 2	
69 ± 2	72 ± 2	89 ± 1	
80 ± 8	80 ± 1		

For each shape, data are ordered from top to bottom corresponding to each SLV image of Fig. 2 from left to right. Values are in percentages.

Geometric effects emerged already for cubic SLVs, where the surface’s principal curvatures are non-uniform and higher at corners and edges. From the equatorial sections of Fig. 2b we see that, at low *x*_LD_, LD domains are predominantly located alongside edges, indicating that softer domains have affinity for regions of higher curvature^9,11–15,17,18^, as a consequence of the lower energetic cost of bending. For higher *x*_LD_ we could not easily identify any pinning to the underlying geometry, as the lack of a complete 3D reconstruction did not allow us to draw further conclusions. Still, about a third of the phase-separated cubes showed three or more domains, indicating that the presence of curvature inhomogeneities competes with interfacial line tension, in such a way as to hinder the coalescence of the lipid domains.

More dramatic curvature effects were seen in symmetric dumbbells, where interface location was almost always correlated to the underlying geometry, as shown in Fig. 2c. For the phase-separated particles with low *x*_LD_ (below  ~23%) that we collected, the POPC-rich phase was indeed pinned to the highly curved neck. However, the vast majority (87%) of dumbbells instead had *x*_LD_ ~ 50%, with two different lipid compositions on each spherical lobe and an interface along the small neck-like region. Particles with higher *x*_LD_ value exhibited an interface lying along one of the lobes, somewhat resembling, on a single lobe, the configurations of phase-separated spherical SLVs (Fig. 2a). Interestingly, no particle configuration was ever seen to lie in the range *x*_LD_ = 24–50%. This gap in the diagram is shown as a dashed segment in Fig. 2c.

Although we cannot assess the absolute amounts of lipids within a given phase with our methods, from the dumbbells of Fig. 2c with, *x*_LD_ ~ 50%, we infer that there must be some mechanism keeping the interface at the neck, at the expense of changing the local composition of the membrane. Variability in phase composition is a common feature of ternary systems: i.e. different points in the Gibbs phase diagram belong to different tie-lines. This effect alone, however, cannot explain the different concentrations on the dumbbell lobes, which would have otherwise occurred also on spheres and cubes. Therefore, our results suggest that the gap originates directly from the curvature of the colloidal substrate, as if the membrane could adapt its tie-line to accommodate the interface in a specific location.

**Fig. 3:**
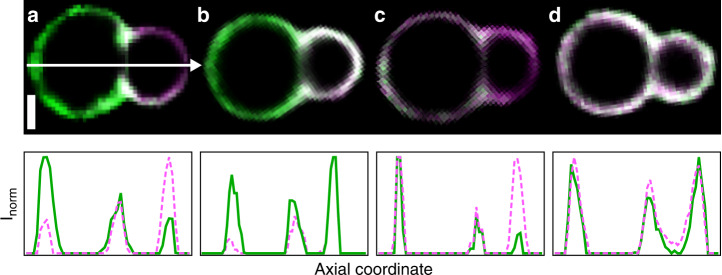
**Different membrane states**. Equatorial plane images (Top) and intensity profiles (Bottom) for snowman-shaped particles. The fluorescence intensity is sampled along the symmetry axis indicated by the white arrow in the top-left image, and normalized over a channel's maximum. Normalized intensities of the green (LO) and magenta (LD) channel are represented by continuous and dotted lines, respectively. The figures shows examples for the four possible configurations: in **a** green and magenta peaks are on opposite lobes representative of the phase-separated state. In **b** and **c**, the peaks are on the same lobe but the intensity is not uniform over the membrane. **d** shows a uniform membrane composition with similar intensities along the whole axis, indicative of the mixed state. Scale bar 2 μm.

The gap in the composition diagram is further enhanced in the case of the snowman particles (see Fig. 2d), which feature an additional curvature asymmetry between the dumbbell lobes. Such phase-separated SLVs exhibit three domains for *x*_LD_ ≲ 13%, with the LD phase located along the neck, and two above this value. In the latter case, the interface lies along the neck and lobe compositions vary continuously, producing a gap that extends all the way to *x*_LD_ = 100%. No other membrane conformations were observed in this large range of *x*_LD_. We quantitatively investigate this striking effect in more detail in Fig. 3. To do so, we measured the intensity of the two fluorescent channels normalised with their maximum intensity along the particle’s long axis and found that some segregated configurations do not have an obvious interpretation. In a phase-separated state, the LO and LD phases produce intensity maxima on different regions of the membrane (see Fig. 3a) as expected. In a mixed state (Fig. 3d), the maxima overlap on both lobes indicating that the lipids are mixed on the whole surface. However, in the configurations displayed in Fig. 3b, c, the SLVs exhibited intensity maxima on the same lobe, while differing in the relative intensity on the other lobe. The membrane regions with different and similar relative intensities can be located both on the smaller or larger lobe of the dumbbell, but on either lobe the composition is always uniform. These quantitative measurements corroborate our hypothesis that geometry affects the distribution of the lipids.

This latter feature shares resemblances with the notion of curvature-driven lipid sorting, as observed in micro-manipulated multicomponent GUVs in proximity of the demixing point^15^. In these experiments, a nanometer-sized membrane tube was pulled from a GUV comprising a ternary mixture of BSM, DOPC, and cholesterol. Despite being in the mixed phase, the striking morphological difference between the tube and the vesicle was observed to affect the local lipid composition, thus driving the membrane away from the homogeneous configuration by a small, yet detectable amount. This effect was ascribed to a tradeoff between mixing and bending energy, leading to an exclusion (enrichment) of those lipids with a tendency to form more (less) rigid bilayers. As we will detail in the following Section, the phenomenon observed here, while originating from the same tradeoff, differs from lipid sorting in two aspects: (1) it does not require the system to be on the verge of demixing; (2) the resulting inhomogeneity in lipid composition is dramatic, spans the whole membrane, and corresponds to membrane compositions lying at antipodal (opposite) sides of local miscibility gaps (i.e. the region in the phase diagram where the mixed phase in unstable to phase separation, see e.g. ref. ^32^). For these reasons, we have termed this regime antimixing in ref. ^33^.

### Antimixing in anisotropic SLVs

Our experiments reveal a correlation between shape and local chemical composition in dumbbell-shaped SLVs: lipids tend to cover uniformly the spherical lobes leaving the interfacial region along the neck, with the POPC molecules clearly preferring lobes of higher curvature. The large variability of relative concentrations (see Fig. 3b, c) implies that it is energetically favourable to have a chemically homogeneous membrane over a single lobe rather than accommodate an interface away from the neck. As explained earlier, this phenomenon cannot be described only by the random spread of SLVs along different tie-lines. We infer that it is the bilayer shape that influences the thermodynamic stability of the lipid mixture.

In ref. ^33^ we had theoretically predicted this scenario with the help of a phase-field model featuring explicit couplings between the lipid chemical compositions and geometry. In the following, we will explain how this approach can elucidate the observed gaps in the experimental phase diagram in the tractable case of a binary mixture, i.e. a membrane consisting of only two different types of molecules. Therefore, the following discussion is to be intended as a qualitative explanation of the underlying phenomena in SLVs, keeping in mind that a quantitative understanding is possible only when considering ternary systems.

A thermodynamically closed system consisting of two incompressible species, say *A* and *B*, can be described by a single scalar order parameter *ϕ*, representing the relative concentration of either one of the species, e.g.:

1 $$\phi =\frac{[A]}{[A]+[B]}\ .$$

By construction, a phase consisting exclusively of type-*A* molecules has *ϕ* = 1, whereas a phase consisting exclusively of type-*B* molecules corresponds to *ϕ* = 0. Yet, since thermodynamically stable phases are never pure, the concentrations corresponding to the LO and LD phases will strictly lie within the interval 0 < *ϕ* < 1. The Helmholtz free energy of the binary system can then be written as:

2 $$F=\int_{\Sigma }{\rm{d}}A\ \left[\frac{D}{2}{\left|\nabla \phi \right|}^{2}+f(\phi )+k(\phi ){H}^{2}+\bar{k}(\phi )K\right]\ ,$$

where the integral is extended over the mid-surface Σ of the lipid bilayer. *H* and *K* are, respectively, the mean and Gaussian curvatures of Σ and we assume that the substrate and PEG molecules do not significantly affect the bilayer symmetry, allowing us to ignore spontaneous curvature effects. The homogeneous part of the free energy density is *f*(*ϕ*) = *u*(*ϕ*) − *Ts*(*ϕ*), with *u*(*ϕ*) the internal energy and *s*(*ϕ*) the entropy densities. The constant *D* is the area compressibility coefficient, whereas *k*(*ϕ*) and $\bar{k}(\phi )$ are the concentration-dependent bending rigidity and Gaussian splay modulus (see e.g. ref. ^32^). To make the *ϕ*-dependence of the elastic moduli explicit, we introduce a microscopic model of molecular interactions. Using a simple lattice-gas model with Ising-like interactions, minimally coupled with the background geometry, yields *u*(*ϕ*) = *Jϕ*(1 − *ϕ*), $s(\phi )=-{k}_{B}[\phi \mathrm{log}\,\phi +(1-\phi )\mathrm{log}\,(1-\phi )]$, *k*(*ϕ*) = *L*_*k*_*ϕ* and $\bar{k}(\phi )={L}_{\bar{k}}\phi$, with *J*, *L*_*k*_, and ${L}_{\bar{k}}$-independent constants expressing, respectively, the strength of molecular interactions and the propensity of a molecule to adapt to the local mean (*L*_*k*_) and Gaussian (${L}_{\bar{k}}$) curvature. Thus, if ${L}_{k}={L}_{\bar{k}}=0$, the molecules are insensitive to the curvature of their local environments. On the other hand, if *L*_*k*_ > 0 and ${L}_{\bar{k}}\,> \, 0$, type*-A* (type*-B*) molecules are depleted from (attracted by) regions having nonvanishing mean curvature and positive Gaussian curvature. For other choices of couplings, see ref. ^33^ and references therein.

**Fig. 4:**
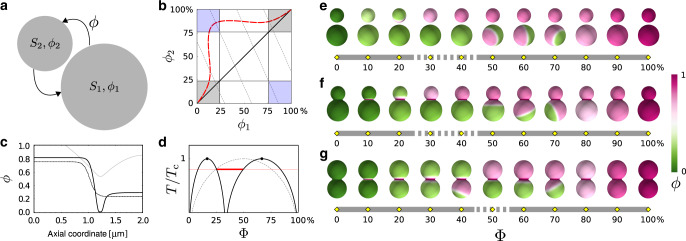
**The antimixed state**. **a** Schematic representation of the two-spheres approximation of the snowman particle of Fig. 2d. **b** Equilibrium lines in the absence (solid black) and presence (dashed red) of curvature coupling *L*_*k*_ in the energy Eq. (2). The vertical and horizontal background lines correspond to the binodal concentrations on each sphere. Dashed oblique lines correspond to fixed values of Φ. Grey squares corresponds to regions where homogeneous mixing and lipid sorting (in the sense of ref. ^15^) can happen. Blue squares highlight regions where antimixed states are global equilibria. **c** Local concentration *ϕ* as a function of the arc-length axial coordinate for the snowman particle at Φ = 0.35. The solid and dashed lines correspond, respectively, to the antimixed state (see Fig. 4f) and to standard phase separation (see Fig. 5b). While the standard phase-separated state is the typical monotonic kink-like curve interpolating between two binodal concentrations, in the case of antimixing the curve is not monotonic and interpolates between average concentrations on the spheres that are precisely the ones predicted by the two-spheres model. The thin grey curve in the background shows, for reference, the shape profile of the snowman geometry. **d** Composition-temperature phase diagram for the two-sphere system when ${L}_{k}=0.14{T}_{{\rm{c}}}{R}_{1}^{2}$, with *R*_1_ the radius of the largest sphere. The binodal line of the classical picture (dashed grey) splits into two lines which determines sub-diagrams where phase separation occurs on either one of the two spheres. The red line corresponds to *T* = 0.9*T*_c_, used in the numerical simulations. The thicker red segment locates the range of the antimixed state for that temperature. **e** Numerically found concentration diagrams of the two spheres at *T* = 0.9*T*_c_. Note that at Φ = 0, 1 also *x*_LD_ = 0, 1, but otherwise the two values do differ. For Φ ~ 0.3−.4 (dashed grey bar) we indeed observe antimixed states. **f**, **g** Numerically found concentration diagrams for the connected snowman and dumbbell geometries. In this case both antimixed states (dashed grey bar) and the geometric pinning along the neck are observed.

The free energy Eq. (2) can now be minimized for all experimental geometries, subject to the constraint of constant area fractions: i.e. Φ = 1/*A*∫d*A* *ϕ* is a fixed quantity. Note that Φ corresponds to the fraction of the total available area occupied by type*-A* molecules and approximately equates *x*_LD_ only in the presence of phase separation. To shed light on the gaps observed in the concentration diagrams of Fig. 3c, we consider a simplified geometry where snowman particles are approximated by two disjoint spheres of radii *R*_1_ > *R*_2_, allowed to exchange molecules among each other (see Fig. 4a). For this system we can formulate the problem in terms of the two average concentrations, *ϕ*_1_ and *ϕ*_2_, on each sphere. By varying the total concentration Φ, a series of points in the {*ϕ*_1_, *ϕ*_2_} plane can be obtained, as we show in Fig. 4b. These determine the equilibrium state reached by the whole system. In absence of an explicit coupling with the curvature (black line in Fig. 4b), the mixed state *ϕ*_1_ = *ϕ*_2_ is reached. By contrast, if *L*_*k*_ ≠ 0, the energetic cost of having *ϕ* ≠ 0 on a sphere depends on its diameter, leading to a displacement of the equilibrium line (dashed red line in Fig. 4b). It is possible to prove that these states are stable against phase separation, and thus are true minima of the free energy^33^.

This phenomenon becomes more intuitive when considering the concentration-temperature phase diagram of the mixture. The classical case of the lattice-gas model with conserved order parameter is shown by the dashed background line in Fig. 4d: there is one critical point and, for Φ and *T* values that lie above the binodal line (shown as a thick black line), the homogeneous mixing is the thermodynamic equilibrium. When switching on the coupling with curvature, the binodal splits the into multiple sublines with a local critical point for each sphere. The region above the binodals still corresponds to mixed states which, however, become nonhomogeneous for subcritical temperatures. The portion of the phase diagram below the critical temperature and containing the thick red line is where antimixed states are stable.

Figure 4e shows the concentration diagram, at *T* = 0.9*T*_c_, of the two spheres, corresponding to the red dotted line in Fig. 4d. In such an idealized system consisting of two disconnected spheres, antimixing occurs for Φ ~ 0.3–0.4 and is characterized by a spectrum of equilibrium configurations reminiscent of those found in the experiments (Fig. 2d). To demonstrate that antimixing is not an artefact of disconnected geometries, we have further considered the cases of fully connected snowmen and dumbbell particles (Fig. 4f, g). The average concentration on the two lobes follows the same equilibrium line of Fig. 4b, and thus, for approximately the same values of Φ as for the disconnected spheres, the equilibrium state is antimixed. Antimixing therefore is characterized by significantly different compositions on the two lobes that, individually, would correspond to a mixed state. Hence, the antimixed state is not a thermodynamically independent phase, but rather a geometry-induced distinct realization of a mixed phase. This is better shown by the concentration profiles of Fig. 4c, where the antimixed configuration in proximity of the neck of a snowman particle (solid black line) is compared with a phase-separated configuration (dashed black line). Whereas the latter follows the classic hyperbolic tangent profile, the antimixed configuration interpolates non-monotonically between non-binodal values in the two lobes. Note that these profiles are nontrivial and cannot be attained analytically, in contrast to the two-spheres model of Fig. 4a, b.

Finally, at high (low) Φ values, the two lobes of snowmen particles, are both in a nearly homogeneous mixed state, where, however, the intensity of magenta (green) on the two lobes is slightly different. For the case of two disconnected spheres, these configurations are highlighted by light grey regions in Fig. 4b, whereas the antimixed state is marked in light blue. These slightly inhomogeneously mixed configurations are the exact analog to the lipid-sorted configurations observed in membrane tubes^15^ and, as anticipated in the experimental results, are well distinct from the antimixed state, although both phenomena originate from the tradeoff between bending and mixing energy. We note that to observe antimixing two portions of the membrane with different curvature yet relatively similar area are required: for dumbbells with very different lobe radii, this effects entirely disappears (Supplementary Fig. 10).

### Geometric pinning and estimation of elastic parameters

As detailed in the experimental results, there exists a large range of concentrations where our SLVs are organized in two or more LO and LD domains. Their location is pinned in specific positions on the substrates, in such a way as to reduce the amount of bending of the stiffer LO phase at the expense of the softer LD phase. In this section, we use these geometrically pinned configurations to extract information about the elastic moduli of the lipid bilayer.

Within the phase-field framework, phase separation can be described using the same free energy of Eq. (2), whose parameters, however, are adjusted in such a way that the corresponding minimizers are now well inside the miscibility gap. Following a standard approach (see e.g. refs. ^34,35^), this can be achieved by taking *D* ~ 1/*f*(*ϕ*) ~ *ξ*, where *ξ* < 1 is a small dimensionless number controlling the thickness of the interface, which now has the typical hyperbolic tangent profile displayed in Fig. 4c. In the limit *ξ* → 0, the diffuse interface shrinks into a line separating the two phases. Furthermore, to avoid computational stiffness, we expand *f*(*ϕ*) at the fourth order in *ϕ* in such a way as to recover the classic double-well potential favouring configurations with *ϕ* = 0, 1. Thus *f*(*ϕ*) = *f*_0_/*ξ* *ϕ*^2^(1 − *ϕ*). Furthermore, we take *k*(*ϕ*) = Δ*k* *g*(*ϕ*) and $\bar{k}(\phi )=\Delta \bar{k}\ g(\phi )$, where Δ*k* = *k*_LD_ − *k*_LO_ and $\Delta \bar{k}={\bar{k}}_{{\rm{L}}D}-{\bar{k}}_{{\rm{L}}O}$ are the differences in bending and Gaussian splay modulus between the LD and LO phases and *g*(*ϕ*) is a suitable function interpolating between 0 and 1. Here we choose *g*(*ϕ*) = *ϕ*^2^(3 − 2*ϕ*), but its precise form becomes unimportant as *ξ* → 0.

As we demonstrated in ref. ^33^, for this choice of parameters, Eq. (2) converges to the classical Jülicher–Lipowsky free energy, namely:

3 $${\mathrm{lim}} _{\xi \to 0}\;F=\sigma \oint _{\Gamma }{\rm{d}}s+\sum_{i\; =\; {\rm{LD}},{\rm{LO}}}\int_{{\Sigma }_{i}}{\rm{d}}A\ \left({k}_{i}{H}^{2}\;+\;{\bar{k}}_{i}K\right),$$

where Σ_LO,LD_ are the portions of *Σ* occupied by the two phases, Γ = ∂Σ_LO_ = ∂Σ_LD_ is the one-dimensional closed interface separating them and $\sigma =2/3\sqrt{2{f}_{0}}$ is the interfacial tension between LD and LO domains. Furthermore, minimizing Eq. (3) for fixed *x*_LD,LO_ area fractions, yields a force balance condition for Γ^36^

4 $$\sigma {\kappa }_{{\rm{g}}}=\Delta k\ {H}^{2}+\Delta \bar{k}\ K+\Delta \lambda \ ,$$

relating the interface’s geodesic curvature *κ*_g_ to the underlying geometry of the substrate (the mean and Gaussian curvatures on the right-hand side are evaluated on Γ). The constant Δ*λ* = *λ*_LD_ − *λ*_LO_ is a Lagrange multiplier, analogous to a local pressure difference across the interface, introduced to enforce the global constraint on the area fractions.

Equation (4) is the two-dimensional curved space analogue of the Young*–*Laplace equation for liquid-liquid interfaces: on a flat substrate, where both *H* and *K* vanish, its solutions describe a circular droplet of radius *σ*/Δ*λ*, with the Lagrange multipliers effectively working as a lateral pressure differential across the interface. For spherical particles of radius *R*, for which *H*^2^ = *K* = 1/*R*^2^, the equilibrium interface lies along constant geodesic curvature lines, i.e. circles, whose total area is fixed solely by the values of *x*_LO,LD_. Furthermore, a single, non-maximal circle is the most stable interface^36^, consistent with Fig. 2a and the fact that we never observed more than two coexisting domains on spherical SLVs.

**Fig. 5:**
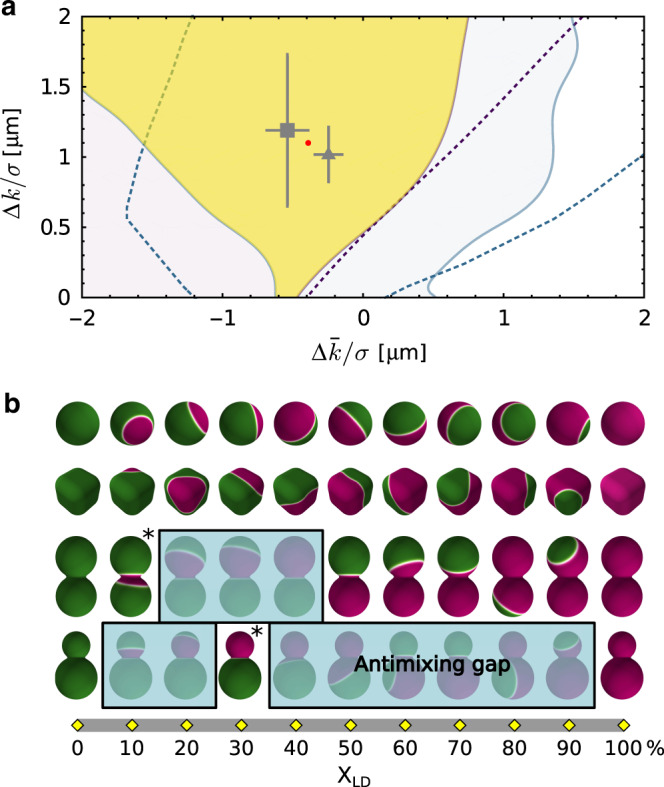
**Elastic moduli differences and interface models**. **a** In the two-dimensional parameter space spanned by ($\Delta k/\sigma ,\Delta \bar{k}/\sigma$), the yellow region shows the parameter values for which the minimal-energy configurations match with the experimental observations for the two SLVs highlighted by the white asterisks in Fig. 2. The solid lines correspond to the boundaries of allowed parameters for the symmetric dumbbell (light blue) and snowman (light magenta) configurations obtained via numerical minimization of Eq. (2), for fixed area fractions. The dashed lines show the analogous result obtained from minimizing the sharp interface model Eq. (4) under the assumption of an axisymmetric interface. For further comparison, we show measurements of these parameters obtained previously using GUVs. The points with error bars are from refs. ^9^ (square) and^10^ (triangle), and their average is shown by a red dot. Error bars are standard deviations of the corresponding data points. **b** Phase-separated configurations for the four geometries used in the experiments obtained from a numerical minimization of Eq. (2) for Δ*k*/*σ* = 1.10 μm and $\Delta \bar{k}/\sigma =-0.39\ \upmu$m, shown as a red dot in **a**. We covered with a blue box the configurations that would correspond to the gaps of Fig. 2. Green and magenta colors correspond to LO and LD phases, and white to the interfacial region). The asterisks show the two configurations that we used to get the yellow region in **a**.

  Figure 5a shows an estimate of the parameters Δ*k*/*σ* and $\Delta \bar{k}/\sigma$ obtained from the sharp interface equation (4) and numerical minimization of the free energy (2), with the choice of parameters given earlier and subject to a constraint on the area fractions. To qualitatively compare our models with experiments, the colloidal shape was extracted from SEM images. We estimate the surface area of the symmetric dumbbell and snowman particles to be 44.6 and 28.4 μm^2^, respectively. Then, using both approaches, we checked for which parameter values we could reproduce the neck-pinned LD domain for the dumbbell at *x*_LD_ ~ 20% and the smaller-lobe-pinned LD domain at *x*_LD_ ~ 30% for the snowman. We labelled these two configurations with a white asterisk in Fig. 2c, d.

The yellow region in Fig. 5a shows the parameter values where numerical results match experimental observations. We found that the two approaches produce qualitatively similar, but not identical results. We ascribe the difference to the different assumptions on the symmetry of the interface. Since the diffuse interface approach relies on fewer assumptions, we consider it to be more reliable.

Interestingly, previous measurements obtained from free-standing vesicles also fall into this region of parameter values for Δ*k*/*σ* and $\Delta \bar{k}/\sigma$^9,10^ (see Fig. 5a). However, we are not able to reach the same accuracy of these works, because SLVs are roughly one order of magnitude smaller in size than GUVs and we cannot optically resolve the precise shape of the neck regions. Furthermore, the substrate does counter-balance any out-of-plane force^35,37^, thus making the structure of the domains less sensitive to small variations of the bending moduli. A consequence of this is that experimental measurements on SLVs are inherently unable to put upper bounds on the model parameters.

## Discussion

In this article, we have introduced an experimental model system to decipher the complex physics of phase mixing and demixing on curved surfaces: scaffolded lipid vesicles (SLVs). The colloidal scaffold imposes its geometry onto the supported lipid membrane allowing an independent study of geometry-induced effects on closed membranes. It is well-known that the LO and LD phases possess different elastic properties and react differently to bending, which induces the geometric pinning of softer LD domains in regions of higher curvature. We observed this effect in SLVs, which allowed us to estimate the LO/LD elastic moduli differences^36^, finding compatible results with previous measures from GUVs experiments^9,10^. Furthermore. the unique combination of closeness and fixed geometry gives rise to antimixing^33^, where lipids segregate in a state, which is continuously connected to a purely mixed phase. This phenomenon is similar in nature to the predicted behaviour of multicomponent lipid membranes on patterned adhesion substrates^38^. In our case the source of inhomogeneity is provided by curvature, and extends the notion of curvature-driven sorting^15^ to membranes with subdomains lying on antipodal sides of local miscibility gaps, from which antimixing takes its name. Using previously developed theoretical tools^33^, we show how the antimixed state can be explained, qualitatively, by a strong influence of curvature on the thermodynamic equilibrium landscape of the lipid mixtures.

While the interplay between geometry and domain localization has been previously observed^9,11–15,17,18^, setting both the shape and the composition of the membrane allowed us to deepen our understanding of the delicate balance between membrane curvature and composition. Although we can qualitatively explain every aspect of our findings, it is evident that a full quantitative description is still missing. This is due to the fact that our experimental system is ternary, and a general understanding of the energy landscape of three-component systems is still lacking. Such a model, thanks to geometry-dependent tie-lines and interface compositions, could explain the full extent of the gaps in the experimental area fraction diagrams.

Our results can be extended to other physical systems exhibiting phase separation^39^. They can provide key insights into how cells regulate local lipid and protein concentrations by adapting membrane shape. Furthermore, the membrane-coated colloids can switch their surface properties and interactions^40^ from uniform to site-specific, paving the way towards smart materials and biomedical applications such as sensing, imaging, and drug delivery. To achieve this goal, control of the lipid composition on single SLV would be highly useful and could be obtained by depositing lipids on colloids with micro-fluidic techniques analogous to studies with emulsions^41^.

## Methods

### Reagents and colloidal dispersions

1-palmitoyl-2-oleoyl-sn-glycero-3-phosphocholine (POPC), porcine brain sphingomyelin (BSM), ovine wool cholesterol (Chol), 1,2-dioleoyl-sn-glycero-3-phosphoethanolamine-N-lissamine rhodamine B sulfonyl 18:1 (Liss Rhod PE), N-[11-(dipyrrometheneboron difluoride)undecanoyl]-D-erythro-sphingosylphosphorylcholine (C11 TopFluor SM), 1,2-dioleoyl-sn-glycero-3-phosphoethanolamine-N-[methoxy(polyethylene glycol)-2000] (DOPE-PEG 2000) were purchased from Avanti Polar Lipids.

Silica spheres were purchased from Microparticles GmbH (2.1 ± 0.1 μm and 7.0 ± 0.3 μm). Polystyrene-3-(Trimethoxysilyl)propyl methacrylate (PS-TPM) dumbbell and snowman particles were synthesised by making a protrusion from swollen polystyrene particles^42^. Hematite cubic particles were made following the method of^43^ and coated with silica^44^. Their corner-to-corner distance was 1.8 ± 0.1 μm. All errors reported for particle sizes are standard deviations.

HEPES buffer was made with 115 mM NaCl, 1.2 mM CaCl_2_, 1.2 mM MgCl_2_, 2.4 mM K_2_HPO_4_, and 20 mM HEPES.

### Lipid bilayer coating

A mixture of 500 μg of BSM, POPC and cholesterol in 2:1:1 mole ratio was prepared in chloroform. 0.2% mole fraction of Liss Rhod PE and C11 TopFluor SM were added to fluorescently label the liquid-disordered and liquid-ordered phases respectively. Five percent mole fraction of DOPE-PEG 2000 was added to improve the mobility of the bilayer. The lipids were dried in 2 h by vacuum desiccation for two hours and then re-suspended to a 2 mg/mL solution with HEPES buffer. The solution was vortexed for 15 min and heated to 70 °C. The lipid solution was extruded 21 times with a mini extruder (Avanti Polar Lipids) equipped with two 250 μL gas-tight syringes (Hamilton), four drain discs and one nucleopore track-etch membrane with pore size 50 nm (Whatman) and placed on a heating plate set at 70 °C. Then, in a rotavapor (Buchi) 50 μL of SUVs were added to 1 mL of 0.05%w/v of particles dispersed in HEPES buffer. The tubes containing the samples were wrapped with aluminium foil to avoid bleaching and placed in a flask filled with water. By keeping the flask in contact with a water bath at 70 °C for 1 h, the samples were mantained above the transition temperature and by gentle rotation, sedimentation of the colloidal particles was avoided. The temperature and the time were chosen to keep the SUVs in the mixed state during the spreading and to give them enough time to spread on the surface of the particles. The solution was then centrifuged at 43 rcf for 10 min and the supernatant replaced with HEPES buffer to remove any SUV in excess.

### Imaging and analysis

The SLVs were imaged at room temperature with an inverted confocal microscope (Nikon Eclipse Ti-E) equipped with a Nikon A1R confocal scanhead with galvano and resonant scanning mirrors. A ×100 oil immersion objective (NA = 1.4) was used. 488 and 561 nm lasers were used to excite excited, respectively, TopFluor and Lissamine Rhodamine dyes. Lasers passed through a quarter wave plate to avoid the polarisation of the dyes and the emitted light was separated by using 500–550 nm and 565–625 nm filters.

Mobility of the bilayer was checked by fluorescence recovery after photobleaching (FRAP) experiments in which the recovery of the signal was fitted with an exponential curve. Examples of FRAP experiments are reported in Supplementary Fig. 4. Three-dimensional image stacks were acquired by scanning the sample in the z direction with a MCL Nano-drive stage and reconstructed with Nikon AR software.

The composition of the SLVs was analysed by using Python scripts. The scripts can be found on GitHub^45^.

Briefly, for each slice of the zeta-stack we applied a threshold to both channels to eliminate random noise and correct, at least partially, the crosstalk between the two dyes. Then we applied a Gaussian threshold and blur to enhance the edges of the membrane. Finally we fitted with 1000 points the intensity profile of the spheres, cubes, dumbbell, and snowman particles to a circle, a squircle, a nephroid and two circles respectively. For the dumbbell shape we compared the quality of the fits by two circles and by a nephroid and chose for the shape from the best fit. From the fit we retrieved the values of the intensity in the magenta and green channels and normalised them with the value of the maximum intensities. We checked along the points of the profile which normalised intensity was higher and

- If *I*_m_ > *I*_g_ we defined that point to belong to the liquid-disordered phase.
- If *I*_g_ > *I*_m_ we defined that point to belong to the liquid-ordered phase.

We then averaged the results over all points of the slice. We found the total area fraction of the image stack by calculating the mean of the line fractions in all slices.

### Details on the numerical simulations

In this Appendix we explain how the 3D geometries corresponding to the experimental SLVs were constructed and how we implemented the numerical solver to find minimal configurations of Eq. (3) and Eq. (2), which were essential to produce Fig. 5 and Fig. 4. For more details see also refs. ^33,46^.

### Gradient flows

The general strategy to find the equilibria of phase-field models is to implement an evolution equation that flows an arbitrary configuration smoothly towards energy minima. Extending previous work^47^ we choose to implement a gradient flow with conserved global order parameter:

5 $${\partial }_{t}\phi =\xi {\nabla }^{2}\phi -\frac{1}{\xi }f^{\prime} (\phi )-k^{\prime} (\phi ){H}^{2}-\bar{k}^{\prime} (\phi )K+\lambda \ ,$$

where ∇^2^ is the Laplace–Beltrami operator on Σ and *ϕ* = *ϕ*(*x*^*i*^, *t*) has been promoted to a function of both space and flow parameter *t*. The right-hand side consists just of (minus) the first functional derivative of the total free energy with a term involving the conservation of the total concentration. When minimizing Eq. (2) one has to replace *k*(*ϕ*) → *k*(*ϕ*)/*ξ* and $\bar{k}(\phi )\to \bar{k}(\phi )/\xi$ in the dynamical equation.

The flow generated by Eq. (5) is fictitious and does not reflect the actual coarsening dynamics of the binary fluid. This approach offers nonetheless a practical way to generate stable equilibrium configurations for arbitrary geometries. Note that *t* has dimension of a length.

The numerical integration of Eq. (5) is done via a local Runge-Kutta-Fehlberg adaptive time-step algorithm^48^, where the criterion for acceptance of a given time-step is averaged over *Σ*.

Metastable equilibrium is defined as the fixed point of the right-hand side of Eq. (5), i.e. when ∂_*t*_*ϕ* vanishes everywhere. Numerically, we assume that the simulation has reached equilibrium when the surface-averaged squared time-derivative

6 $${\left\langle {\left({\partial }_{t}\phi \right)}^{2}\right\rangle }_{\Sigma }=\frac{1}{{A}_{\Sigma }}\int_{\Sigma }{\rm{d}}A{\left({\partial }_{t}\phi \right)}^{2}\ ,$$

decreases below a given threshold (usually of the order of 10^−6^ for surfaces of unit area).

The initial conditions, at *t* = 0, are either random *ϕ* values distributed around a specific Φ value (for Eq. (2)) or a random set of small domains with constant *ϕ* = ϕ_*_ surrounded by a 1 − ϕ_*_ background (for Eq. (3) and to generate the phase diagram of Fig. 5a).

### Mesh construction

We solve Eq. (5) numerically using a finite difference scheme on unstructured triangular meshes. Meshes are constructed using the software package *Gmsh*^49^. As in the case of planar droplets on the plane, the symmetry of the substrate is not necessarily inherited by the minimizers of the Gibbs free energy, thus it is often necessary to solve the full two-dimensional problem. For the case of dumbbells and snowmen, the radial profile has been obtained by joining two circular arcs by an interpolating polynomial of degree eight, chosen such that the neck interpolation and the circular arc match smoothly up to the fourth derivative at each of the two glueing points.

Due to the arbitrariness of the geometry of Σ, our implementation of the solver is entirely coordinate-independent. Computation of discrete Laplace–Beltrami weights and of the mean curvature *H* on each node is done by using the cotangent method. Since the ideal surface is smooth and differentiable at least twice, we use the numerically computed *H* as a criterion for the quality of the triangulation: if *H* appears to be reasonably smooth on every portion of Σ we accept the mesh, otherwise we refine it.

For every triangulation, the length scale in a simulation is fixed by the requirement that the area of Σ has to match the dimensions in μm^2^ of the observed SLVs. Furthermore, the value of *ξ* is automatically computed for each triangulation and is fixed by the requirement that the interface should be resolved anywhere on the membrane by at least six grid points.

### Equilibrium equation in nonhomogeneous potentials

If the free energy depends explicitly on the position through the non-constancy of curvatures, one can divide Σ in regions—each labeled by an index *i*—where both *H* and *K* can be regarded as approximately constant, so that

7 $$F=\sum_{i}{x}_{i}{F}_{i}({\phi }_{i})\ ,$$

where the sum runs over the partition of Σ, *x*_*i*_ is the area fraction occupied by the subregion *i* (so that ∑_*i*_*x*_*i*_ = 1). The variable *ϕ*_*i*_ is the restriction of the field *ϕ* to the region *i*. If Σ is a smooth surface, it is always possible to find partitions that satisfy the above requirement to an arbitrary degree of accuracy. The condition for equilibrium is obtained by differentiating *F* in each subdomain:

8 $$\frac{{\rm{d}}{F}_{i}}{{\rm{d}}{\phi }_{i}}=\mu \ ,$$

provided the average concentration

9 $$\sum_{i}{x}_{i}{\phi }_{i}=\Phi \ ,$$

is kept fixed. Eq. (8) implies that, at equilibrium, the derivative of each free energy has to take the same value over all of Σ. This should not be confused with the usual requirement that, at equilibrium, two coexisting phases have always the same value of the chemical potential: here the free energies are position-dependent functions and Eq. (8) refers to the equilibrium condition of a single, mixed phase at different locations in space. Note that now, for generic curvature dependence, the mixed phase *ϕ*_*i*_ = Φ is not a solution of Eq. (8). If some, or all, *F*_*i*_ happen to be concave, then one should check whether the spinodally decomposed solutions can become locally favoured. We found the solutions of Eq. (8) when Σ consists of the disjoint union of two spheres to produce the equilibrium phase diagrams of Fig. 4b–d. For more details on this specific case see^33^.

## Supplementary information

Supplementary Information

## Data Availability

All data needed to evaluate the conclusions in the paper are present in the paper and the Supplementary Information. Additional data related to this paper may be requested from the authors.

## Code availability

The source code used for measuring the fraction of liquid phases from fluorescence images and for simulating the behaviour of binary mixtures on curved surfaces is available on Github^45,46^.
