# Supplementary material for: Geometric pinning and antimixing in scaffolded lipid vesicles
Source: Nat Commun. 2020 Sep 4;11:4314. doi: 10.1038/s41467-020-17432-w (PMC7474073; doi:10.1038/s41467-020-17432-w)
Supplement: Supplementary file 1 — Supplementary Information [file 41467_2020_17432_MOESM1_ESM.pdf]

Supplementary Information for: Geometric pinning and antimixing in scaffolded lipid vesicles

Melissa Rinaldin, Piermarco Fonda, Luca Giomi, Daniela J. Kraft

Supplementary Methods

Reagents

Sodium hydroxide (NaOH) pellets $\geq$ 97%, iron (III) chloride (FeCl3) hexahydrate puriss. 98-100%, tetraethyl orthosilicate acid (TEOS) 37%, hydrochloric acid (HCl) 37%, potassium chloride (KCl) 99+% and tetramethylammonium hydroxide solution (TMAH, 25% wt) were purchased from Sigma Aldrich. Ethanol puriss. $\geq$ 99.8% and ammonia solution (NH_4OH) 28%-30% were obtained from Honeywell. Silica particles (2.06 $\pm$ 0.05 µm, 7.00 $\pm$ 0.29 µm) were purchased from Microparticles GmbH. Water with 18.2 MΩ cm resistivity obtained from a Millipore Filtration System (Milli-Q Gradient A10) was used in all experiments.

Synthesis and silica coating of colloidal particles with cubic shape

Hematite colloids with a cubic shape were synthesised following the sol-gel method of Sugimoto *et al.* [1]. Specifically, a sodium hydroxide (NaOH) solution was prepared by dissolving 20.14 g of NaOH in 100 mL water. This solution was added in 50 s to a 100 mL solution of 50.39 g iron (III) chloride ($FeCl_3$) dissolved in water while magnetically stirring at 300 rpm. Weighting of the $FeCl_3$ was done quickly because the salt is very hygroscopic. The mixture was stirred for another 10 minutes at 450 rpm and then transferred to a preheated oven at 100 °C, where it was left undisturbed for 10 days. The resulting cubic particles had a superball shape which can be described by the parameter m [2]:

$$m = \frac{\log 2}{0.5 \log 2 - \log \frac{L}{D}} \tag{1}$$

where L and D are respectively the side and the corner-to-corner length of the cubic particle. The hematite cubes obtained from our synthesis had a $m-$value of 3.3 $\pm$ 0.6 and a corner-to-corner distance of 1.76 $\pm$ 0.08 µm. A TEM image of the particles is shown in Figure 1 A. The particles were washed and stored in ethanol.

To coat the particles with a silica layer, we followed a method described by Rossi et al. [3]. The reaction was preformed at 15 - 20 °C in a 2L round bottom flask positioned in an ultrasonic bath (Elmasonic P300H, Elma). The reaction flask contained a uniform mixture of 920 mL ethanol, 62 mL of water, 42 mL of cube dispersion (3.8 %wt), 10 mL tetramethylammonium hydroxide (TMAH) dissolved in water (1% wt). To coat the particles with silica a mixture of 15 mL tetraethyl orthosilicate (TEOS) and 15 ml ethanol was added to the reaction flask under mechanic stirring at a rate of 230 µL/min using a syringe pump (Harvard apparatus). After addition, the reaction mixture was sonicated for 3h and stirred for another 16h to ensure that all TEOS had reacted (Supplementary Figure 1B).

These silica coated hematite cubes were then turned into hollow silica cubes by dissolving the hematite cores in 5M hydrochloric acid. The hollow silica cubes were washed three times and stored in ethanol (Supplementary Figure 1C).

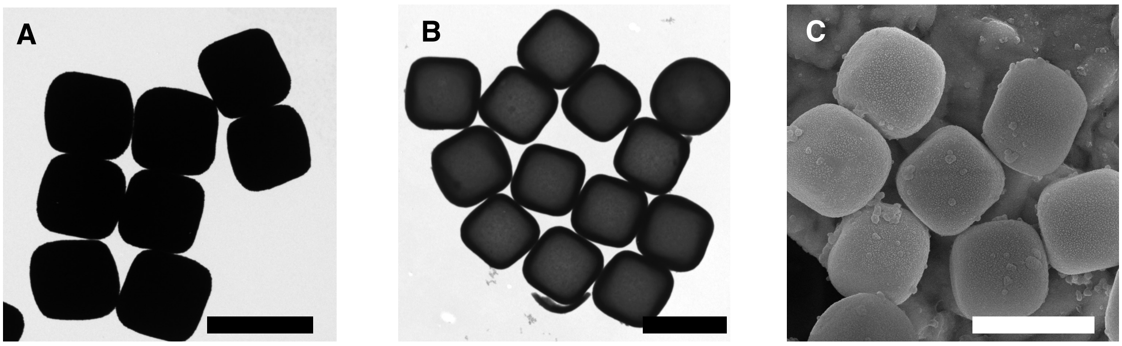

Supplementary Figure 1: **Transmission electron microscopy (TEM) images of colloidal particles. A)** hematite cubes and **B)** silica coated hematite cubes, and **C)** scanning electron microscopy (SEM) image of silica cubes after dissolution of the hematite core. Scale bars 2 µm.

Synthesis and silica coating of dumbbell and snowman-shaped particles

Colloidal dumbbell and snowman particles consisting of polystyrene (PS) and 3-(Trimethoxysilyl)propyl methacrylate (TPM) were synthesised using a modified version of the procedure described by Kim J. *et. al.* [4].

Linear polystyrene particles were synthesised by a dispersion polymerisation method and cross-linked by the addition of a swelling solution containing 90:10 styrene: TPM and divinylbenzene (DVB). After polymerisation initiated by azobisisobutyronitrile (AIBN) a second swelling step was performed to create a protrusion on the cross-linked spheres. Depending on the swelling ratio S, which is defined as the mass of the monomer/the mass of the polymer colloids, dumbbell particles ($S = 3$) or snowman shapes ($S = 4$) were obtained. The dumbbell particles have total length of 5.23 ± 0.05 µm and the ratio between the diameters of the two lobes is equal to 0.98 ± 0.04 (Supplementary Figure 2A). The snowman particles have total length of 4.01 ± 0.04 µm and the ratio between the diameters of the two lobes is equal to 0.57 ± 0.02 (Supplementary Figure 2B).

To achieve adsorption of the lipids to the surface of these particles, the dumbbell particles were coated with silica. We used a modified version of the protocol by Wang et al. to coat hematite particles [5]. Typically, 0.5 mL TEOS was added to an ultrasonicated mixture of 100 mL ethanol, 15 mL ammonia (28%-30%) and 5 mL of particle dispersion (0.5% wt) while mechanically stirring for 5h. The silica coated colloids were washed and stored in ethanol. A SEM image of the resulting dumbbell particles is shown in Supplementary Figure 5B).

Silica dumbbells fabricated by destabilization of silica spheres

To investigate how different silica surfaces affect the phase separation of the bilayer, we prepared silica particles with a dumbbell shape and with a size comparable to the silica-coated PS-TPM particles through an alternative route. Colloidal dumbbell and snowman shaped particles of silica were obtained by destabilisation of charge-stabilised silica spheres. Destabilisation was achieved by mixing a solution of 200 µL potassium chloride (KCl) with 100 µL dispersion of silica particles (2.06 ± 0.05

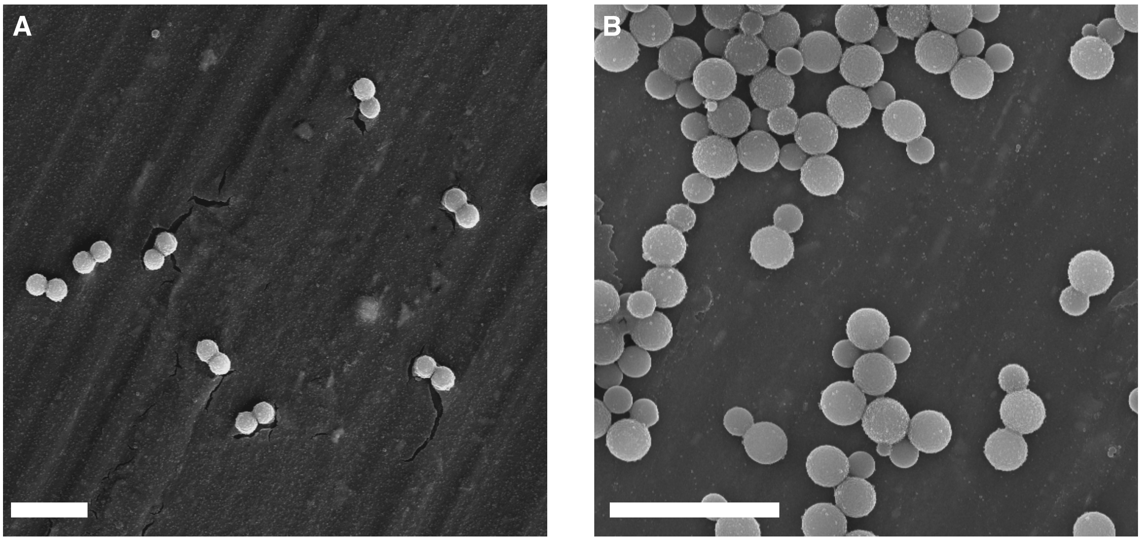

Supplementary Figure 2: **Scanning electron microscopy (SEM) images of colloidal particle. A)** polystyrene (PS) and 3-(Trimethoxysilyl)propyl methacrylate (TPM) dumbbell particles and **B)** PS-TPM snowman particles. Scale bars 10 µm.

µm and 7.00 ± 0.29 µm) in water (5 %wt). The mixture was tumbled end-over-end for 20 minutes before being quenched with 20 mL water. The resulting aggregates consisted of varying numbers of spheres were washed six times with water to remove the KCl and re-stabilise the particles. An example of an obtained dumbbell particle is reported in Supplementary Figure 5A. We coated an aliquot of the dispersion with a lipid bilayer and then only inspected phase separation on aggregates consisting of two spheres.

Fluorescence Recovery After Photobleaching

A fundamental property of the lipid bilayer that allows phase separation is its fluidity, that is the lateral diffusion of the lipids. To check the fluidity of the bilayer we used the Fluorescence recovery after photobleaching (FRAP) technique. FRAP is a method that consists of bleaching a fluorescent area of the sample and observing the fluorescence recovery. In this work we used FRAP to check the mobility of the bilayer in the following way: a circular area of the membrane containing fluorescent DOPE-Rhodamine lipids is bleached and the subsequent recovery of the intensity of this region is measured. We observed that the recovery of the signal is exponential (see Supplementary Figure 4) and therefore fitted the data using the following fit function:

$$I_{\mathrm{norm}}(t) = A\left(1 - e^{-\frac{t-t_0}{\tau}}\right) \; , \tag{2}$$

where $I_{\mathrm{norm}}(t) = I(t)/[I(t=0)I(t)_{\mathrm{ref}}]$ is the measured intensity $I(t)$ normalised with respect to the intensity just before bleaching $I(t=0)$ and corrected for bleaching through measurement of the intensity of a non-bleached reference area, $I(t)_{ref}$. A is the extent of the recovery, $t - t_0$ is the time elapsed since the beginning of the recovery process and τ the recovery time. We report in the following table the values of the parameters obtained from the fit in Supplementary Figure 4:

Shape	A	$1/\tau$ [s^{-1}]	t_0[s]
Sphere	0.75 ± 0.02	0.35 ± 0.04	3.9 ± 0.2
Cube	0.74 ± 0.05	0.07 ± 0.02	1 ± 1
Snowman	0.68 ± 0.02	0.08 ± 0.01	5 ± 1

Table 1: Values of the parameters of the exponential fits shown in Supplementary Figure 4.

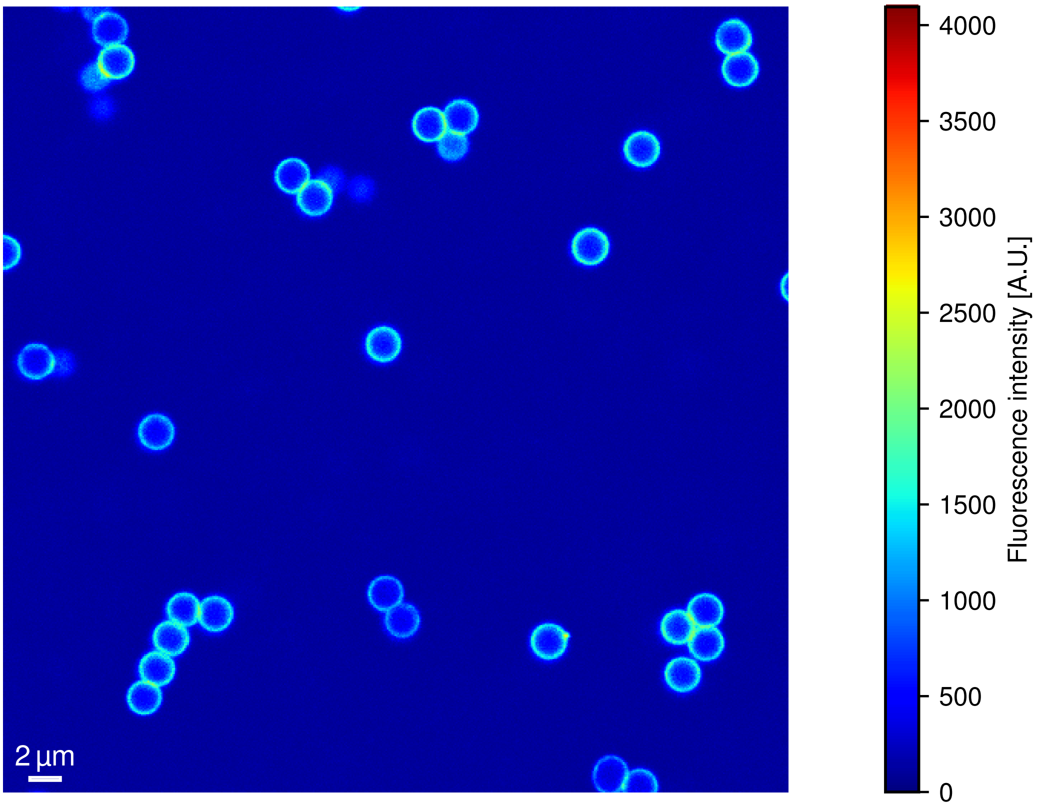

Supplementary Figure 3: **Homogeneity of lipid coating.** To examine that the small unilamellar vesicles (SUVs) spread uniformly on the surface of the particles we prepared spherical SLVs in only the liquid disordered phase, made by mixing 5% 1,2-dioleoyl-sn-glycero-3-phosphoethanolamine-N- [methoxy(polyethylene glycol)-2000] (DOPE-PEG), 94.8% 1-palmitoyl-2-oleoyl-sn-glycero-3-phosphocholine (POPC) and 0.2 % rhodamine B sulfonyl 18:1 (Liss Rhod PE). The fluorescence intensity is uniform across the sample and individual particles indicating that the lipid coating is homogeneous.

Lipid composition

In supported lipid bilayers the substrate can affect the physical properties of phase separation, such as the temperature at which phase separation occurs [6]. Therefore, we varied the lipid composition on spherical supports to identify the conditions under which phase separation occurs. We found that phase separation can be obtained with the following mixtures of porcine brain sphingomyelin (BSM), 1-palmitoyl-2-

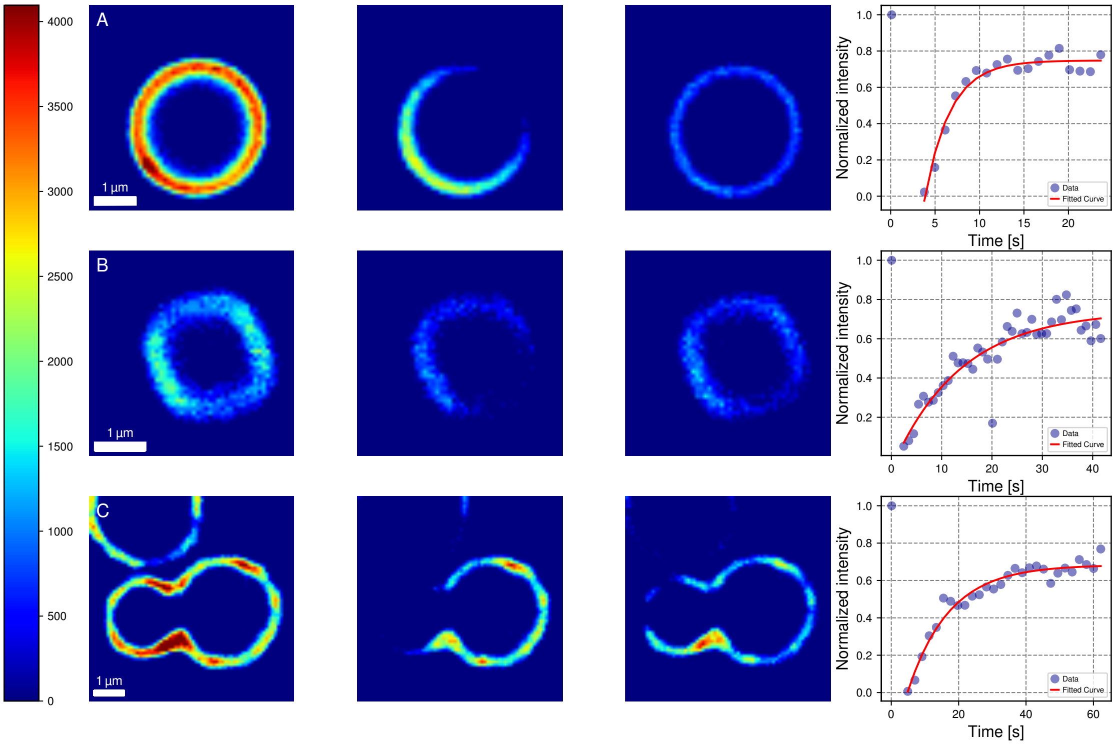

Supplementary Figure 4: **FRAP analysis of SLVs.** **A)** spherical, **B)** cubic and **C)** snowman shaped SLVs. Images were taken at **A)** t=0,4, 25 s, **B)** t=0, 2, 42 s, and **C)** t=0, 5, 62 s. From left to right, confocal microscopy images were taken before, directly after the bleaching and after the full recovery of the fluorescent signal. The fluorescent intensity is colour-coded as shown in the bar on the left. The right column shows the respective data of the normalised intensity and the exponential fit. The parameters obtained from the fit are reported in Table 1.

oleoyl-sn-glycero-3-phosphocholine(POPC) and cholesterol (chol):

- 30% BSM, 50% POPC, 20% chol. mole ratio.
- 35% BSM, 45% POPC, 20% chol. mole ratio.
- 40% BSM, 35% POPC, 25% chol. mole ratio.
- 50% BSM, 20% POPC, 30% chol. mole ratio.
- 50% BSM, 25% POPC, 25% chol. mole ratio.

No phase separation was observed for 0 to 10% of cholesterol on spherical SLVs. We used the mixture 2:1:1 BSM:POPC:chol. This ratio has also been shown to phase separate in free-standing bilayers both at 23°C and 37°C, albeit with a different type of sphingomyelin, the palmitoylsphingomyelin PSM [7]. We confirmed that also for our mixture phase separation occurs in free standing bilayers (giant unilamellar vesicles) made via electro-swelling (see Supplementary Figure 5).

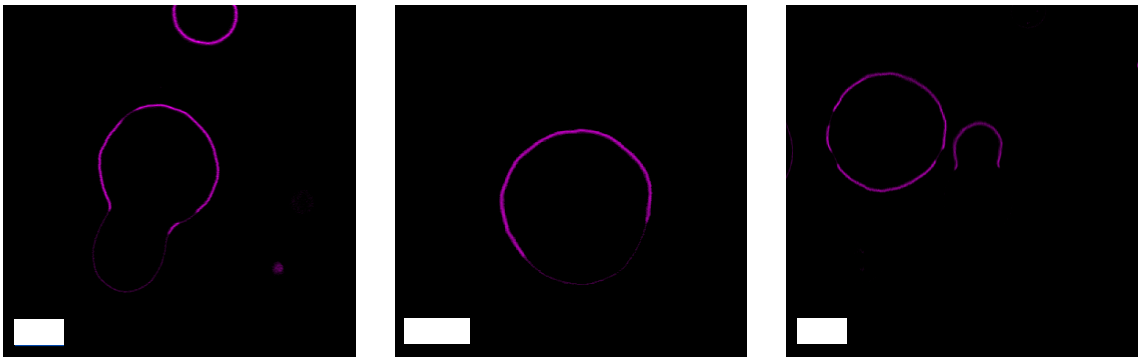

Supplementary Figure 5: **Fluorescence images of phase-separated free standing lipid bilayers.** Lipid composition 2:1:1 SM:POPC:chol. The liquid disordered phase is labelled in magenta with DOPE-Rhodamine. Scale bars 10 µm.

Comparison of probability of phase separation and number of domains on dumbbell particles with different surfaces

In order to exclude that surface properties of the supporting colloidal particles, such as the roughness or the type of silica, affect the phase separation landscape, we compared dumbbell shaped SLVs made on two different types of dumbbell-shaped substrates: silica dumbbells made via destabilisation of colloidal silica spheres and silica-coated dumbbells made from polystyrene 3-(Trimethoxysilyl)propyl methacrylate Silica coated (PS-TPM-Si) particles particles. We studied the likelihood of phase separation and of the number of domain (Figure 7). We observed that these quantities are similar for the two types of particles, indicating that the properties of the surfaces of the supports that we use do not significantly influence phase separation properties.

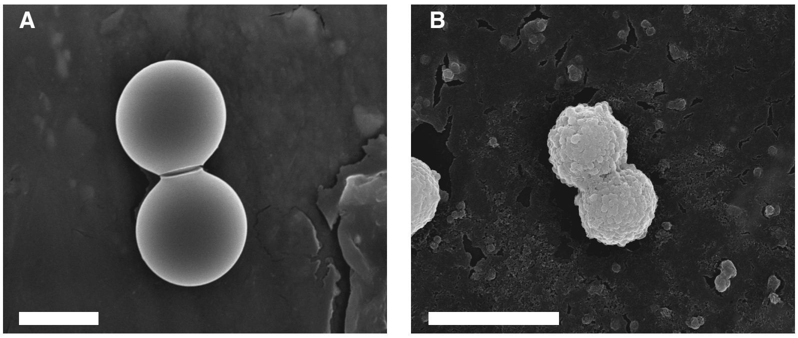

Supplementary Figure 6: **Scanning electron microscopy image of colloidal scaffolds.** **A**) Scanning electron microscopy (SEM) image of a silica dumbbell made via destabilisation of silica spheres. **B**) SEM image of a polystyrene 3-(Trimethoxysilyl)propyl methacrylate Silica coated (PS-TPM-Si) dumbbell. Scale bars 5 µm.

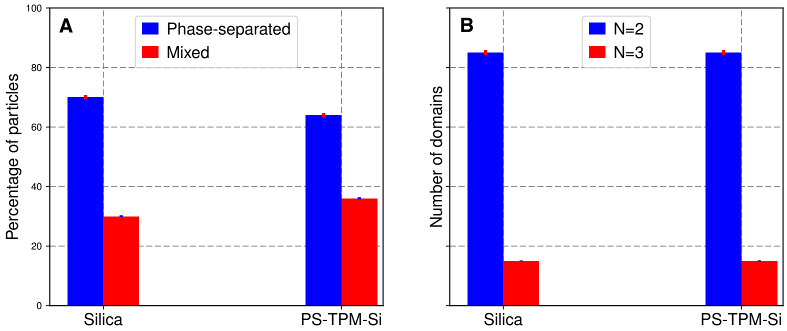

Supplementary Figure 7: **Likelihood of lateral segregation on SLVs.** **A**) Percentage of phase-separated SLVs made of aggregated silica spheres (left) and polystyrene 3-(Trimethoxysilyl)propyl methacrylate silica (PS-TPM-Si) (right). **B**) Histogram of the number of coexistent liquid-ordered and disordered domains of the same SLVs.

Supplementary figures

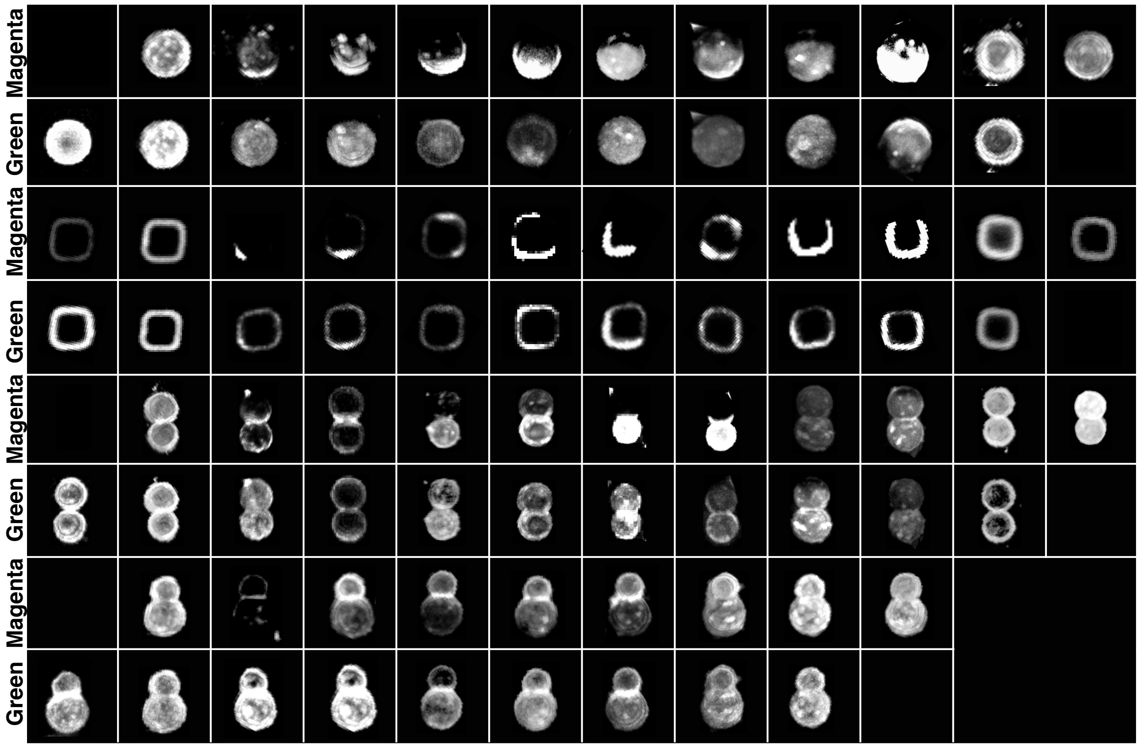

Supplementary Figure 8: **Separate channel images referring to the colour-merged images of Figure 2.** The images are ordered from left to right by amount of area fraction going into the LD phase. Magenta (561 nm) and green channels (488 nm) of the 3D reconstruction (for spherical, dumbbell and snowman shaped SLVs) or the equatorial plane (for cubic SLVs) of the images shown in Figure 1. Scale bar 2 µm.

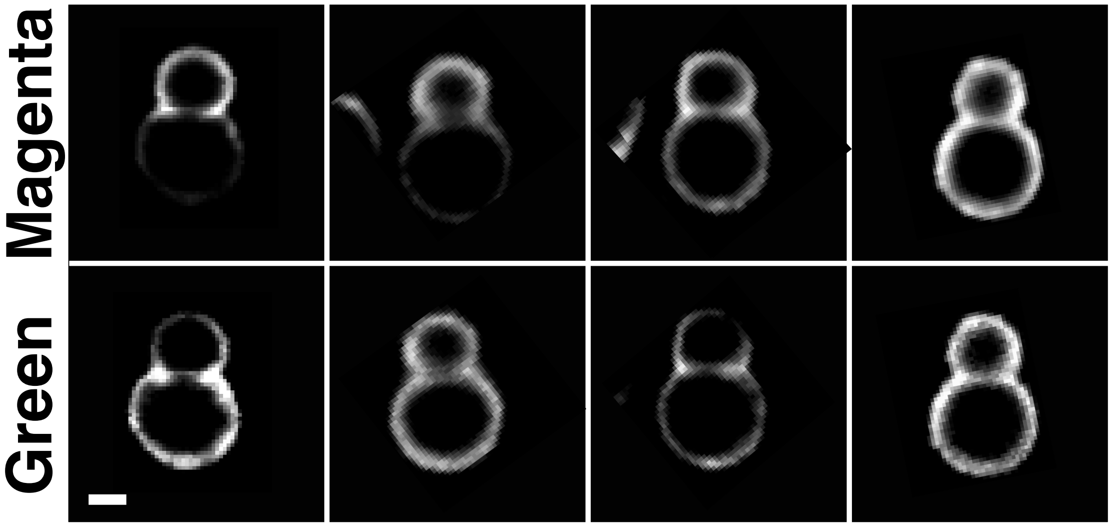

Supplementary Figure 9: **Separate channel images referring to the colour-merged images of Figure 3.** From left to right: a phase-separated state, two antimixed states, and one mixed state. Magenta and green channels of the snowman shaped SLVs shown in Figure 3. Scale bar 1 µm.

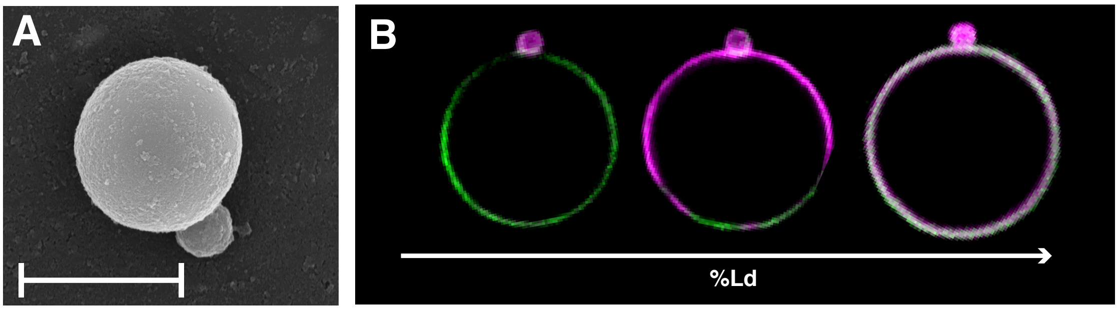

Supplementary Figure 10: **A** SEM image of a cluster of silica spheres. **B** Fluorescence images of SLVs on the colloids in A ordered by concentration of lipids going into the LD phase. From left to right: SLV showing the soft phase and the rigid phase in the smaller and larger lobe, respectively, SLV showing the softer phase in the smaller lobe and phase separation in the larger lobe, and SLV showing the LD phase in the smaller lobe and the mixed phase in the large lobe. In the last configuration, the concentration of lipids going into the LD phase is similar between the two lobes indicating that the SLV shows similarities to the curvature-induced lipid sorting observed in pulling experiments from giant unilamellar vesicles [8]. Scale bar 7 µm.

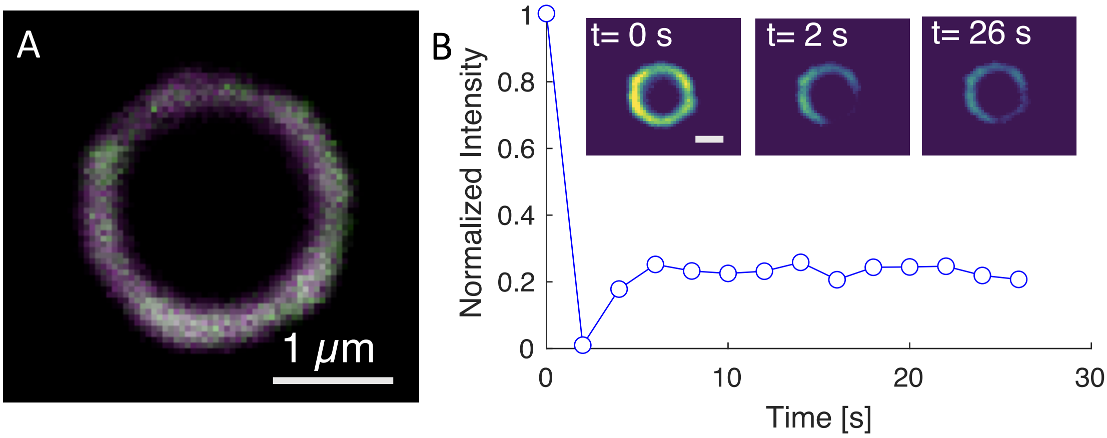

Supplementary Figure 11: **A** Phase-separated lipid bilayer on colloid made with SUVs kept at room temperature. The two phases are localized in random patches on the surface of the colloids and the membrane appeared to have varying thickness, pointing to incomplete fusion of the SUVs on the surface, which prohibits attainment of an equilibrium state. **B** Fluorescence recovery after photobleaching experiment of a colloid made with SUVs kept at room temperature. The dye bleached is the DOPE-Rhodamine. On the top, fluorescence images are taken before the bleaching, after the bleaching, and at the end of the experiment. On the bottom, the normalized intensity corrected for bleaching is plotted with time.

Supplementary references

[1] Sugimoto, T. & Sakata, K. Preparation of monodisperse pseudocubic α-Fe2O3 particles from condensed ferric hydroxide gel. *J. Colloid Interface Sci.* **152**, 587–590 (1992).

[2] Meijer, J.-m. Colloidal Crystals of Spheres and Cubes in Real and Reciprocal Space. *Springer theses* (2015).

[3] Rossi, L. *et al.* Cubic crystals from cubic colloids. *Soft Matter* **7**, 4139–4142 (2011).

[4] Kim, J.-W., Larsen, R. J. & Weitz, D. A. Synthesis of Nonspherical Colloidal Particles with Anisotropic Properties. *J. Am. Chem. Soc.* **128**, 1437414377 (2006).

[5] Wang, Y., Su, X., Ding, P., Lu, S. & Yu, H. Shape-controlled synthesis of hollow silica colloids. *Langmuir* **29**, 11575–11581 (2013).

[6] Chemburu, S., Fenton, K., Lopez, G. P. & Zeineldin, R. Biomimetic silica microspheres in biosensing. *Molecules* **15**, 1932–1957 (2010).

[7] Petruzielo, R. S., Heberle, F. A., Drazba, P., Katsaras, J. & Feigenson, G. W. Phase behavior and domain size in sphingomyelin-containing lipid bilayers. *Biochim. Biophys. Acta, Biomembr.* **1828**, 1302–1313 (2013).

[8] Sorre, B. *et al.* Curvature-driven lipid sorting needs proximity to a demixing point and is aided by proteins. *Proc. Natl. Acad. Sci. U.S.A.* **106**, 5622–5626 (2009).
